# Postoperative Airway Obstruction by a Bone Fragment

**DOI:** 10.1155/2017/4381819

**Published:** 2017-03-30

**Authors:** Patrick Schober, K. Hakki Karagozoglu, Stephan A. Loer, Lothar A. Schwarte

**Affiliations:** ^1^Department of Anesthesiology, VU University Medical Center, De Boelelaan 1117, 1007 MB Amsterdam, Netherlands; ^2^Academic Centre for Dentistry Amsterdam (ACTA) and Department of Oral and Maxillofacial Surgery/Oral Pathology, VU University Medical Center, De Boelelaan 1117, 1007 MB Amsterdam, Netherlands

## Abstract

Postoperative airway obstructions are potentially life-threatening complications. These obstructions may be classified as* functional* (sagging tongue, laryngospasm, or bronchospasm),* pathoanatomical* (airway swelling or hematoma within the airways), or* foreign body-related*. Various cases of airway obstruction by foreign bodies have previously been reported, for example, by broken teeth or damaged airway instruments. Here we present the exceptional case of a postoperative airway obstruction due to a large fragment of the patient's maxillary bone, left accidentally in situ after transoral surgical tumor resection. Concerning this type of airway obstruction, we discuss possible causes, diagnosis, and treatment options. Although it is an exceptional case after surgery, clinicians should be aware of this potentially life-threatening complication. In summary, this case demonstrates that the differential diagnosis of postoperative airway obstructions should include foreign bodies derived from surgery, including tissue and bone fragments.

## 1. Introduction

Postoperative airway obstructions are potentially life-threatening complications. The obstruction can be classified as* functional* (sagging tongue, laryngospasm, or bronchospasm),* pathoanatomical* (airway swelling or hematoma within the airways), or* foreign body-related*. Various cases of airway obstruction by foreign bodies have been reported. Here we present a previously unreported case of postoperative airway obstruction due to an unrecognized fragment of the patient's maxillary bone.

## 2. Case Description

**Figure 1:**
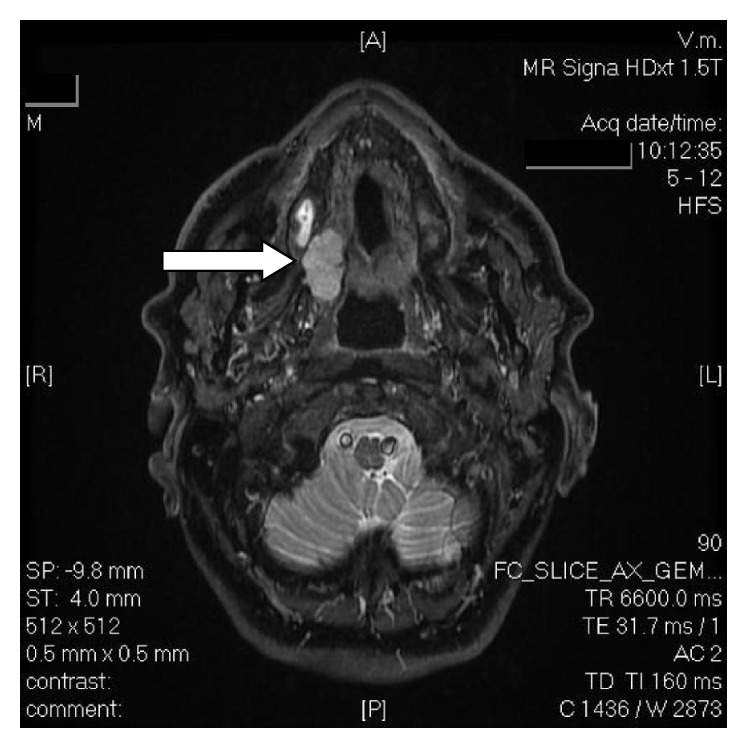
Preoperative horizontal MRI scan indicating the location (white arrow) of the adenocarcinoma of the right palatomaxillary region. [L]: left side; [R]: right side; [A]: anterior aspect; [P]: posterior aspect.

A 74-year-old, male ASA-III patient presented with an adenocarcinoma of the right palatomaxillary region (Figure 1) and was scheduled for maxillectomy for tumor excision and placement of an obturator prosthesis. Preoperative workup revealed a history of smoking, COPD (under therapy with fluticasone, tiotropium, and a budesonide-formoterol combination), mild aortic insufficiency, and an incomplete right bundle branch block on the ECG.

Premedication (oxazepam), induction (propofol, sufentanil, and rocuronium), and maintenance (sevoflurane, N_2_O) of anesthesia were uneventful. The airway was secured by endotracheal intubation via the nasal route to allow optimal transoral access to the posterior maxilla. The pharynx was packed with gauze using a Magill-type forceps to scavenge intraoperative blood and tissue debris, which otherwise could enter the larynx or esophagus.

At the end of surgery, the pharyngeal tamponade was removed and the surgeon rechecked the surgical site for adequate hemostasis. Thereafter, administration of sevoflurane and N_2_O was stopped and the patient was allowed to awaken from anesthesia. With the surgical site being the upper airway, we extubated the patient only after complete return of protective airway reflexes, in particular after occurrence of effective coughing. After extubation, the patient was transferred to the postanesthesia care unit (PACU). When the patient was fully awake, he started to cough, accompanied by tachycardia and arterial hypertension. He was moderately agitated and reported dyspnea and intense pain in his throat, which was attributed to airway irritation following surgery, pharyngeal tamponade, and endotracheal intubation (Table 1). Treatment included administration of analgesia (piritramide i.v. in doses of 2.5 mg) and inhalational nebulizer therapy (with salbutamol and ipratropium bromide). To prevent rebleed in the coughing patient, he received tranexamic acid (500 mg loading dose, followed by 1500 mg i.v. over 15 min).

**Figure 2:**
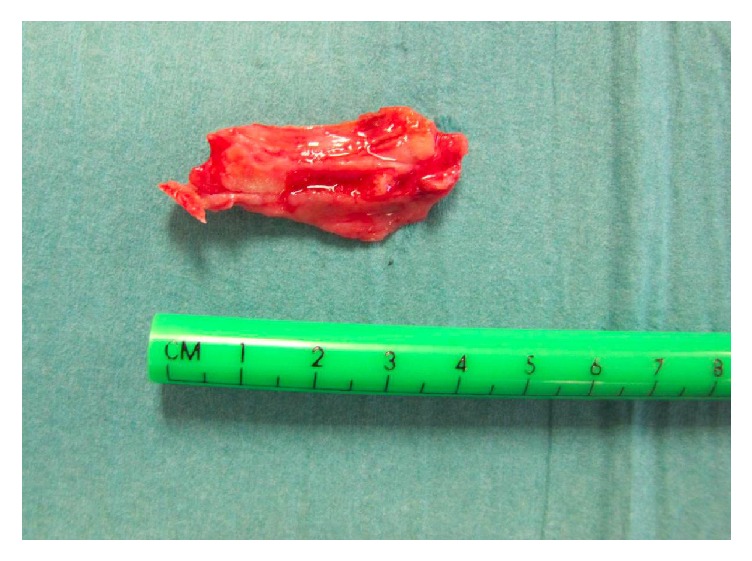
The expelled bone fragment and a cm-scale. The bone fragment has the dimension of 4 × 2 cm.

About 45 min after admittance to the PACU, the patient still presented regular series of coughing. During such a cough attack, the patient suddenly expelled a considerably large, bloody mass. Directly hereafter, frequency and intensity of coughing decreased and the feeling of dyspnea and throat pain disappeared. In parallel, the vegetative stress symptoms like tachycardia and hypertension diminished (Table 1). At closer inspection of the expelled bloody mass, we identified a clot-covered bone fragment of about 4 × 2 cm size (Figure 2). Analysis of the bone at the university pathology institute confirmed that the bone derived from the maxilla.

## 3. Discussion

Perioperative respiratory complications are relatively frequent, with a potentially life-threatening subgroup being postoperative airway obstructions [1], particularly in patients with surgery at the airways. Here we present the case of a foreign body resulting from surgery itself by an intraoperatively “separated” bone fragment. Airway obstruction by a bone fragment was not included in our primary differential diagnosis.

In our literature review on foreign body-induced postoperative airway obstruction, we found cases for various objects left in situ to cause airway problems in the postoperative period. From an anesthesiological perspective, teeth broken during intubation might remain in the airway and even migrate deeper into the tracheobronchial tree [2]. From the devices used intraoperatively, reported objects causing airway obstruction include damaged intubation aids [3], displaced airway packings [4], or forgotten gauze packs [5].

Pharyngeal tamponades with gauze packing are controversially discussed in their efficacy to scavenge intraoperative blood and debris from the surgical wound and thus in their efficacy to seal the esophagus (to prevent emesis) and the larynx (to prevent laryngospasm and airway obstruction) from blood and surgical debris. In the presented case, evidently, the pharyngeal tampon was an insufficient measure to scavenge the large bone fragment. On the other hand, forgetting to remove the pharyngeal tamponade after surgery may itself result in severe airway obstruction and even death.

We add the presented case of a large surgery-derived bone fragment to the list of foreign bodies causing postoperative airway obstruction. In contrast to the clearly defined, inventoried set of surgical instruments used or the known number of gauzes used intraoperatively, there is usually no registration regarding removed tissue and bone pieces intraoperatively. The amount of removed tissue is difficult to quantify and varies from operation to operation. This is of particular importance when tissue is intraorally separated from the patient.

Regarding postoperative airway management, the anesthesiologist should be aware of the different presentations of airway obstruction, for example, inspiratory or expiratory stridor, depending on the level of airway obstruction, to allow proper diagnosis and prompt therapy. In case that the predominant clinical symptom accompanying dyspnea is persistent coughing and (unexpected) airway related pain, the possibility of airway obstruction by a foreign body should be considered and checked by direct inspection or endoscopy, also in the immediate postoperative period. In contrast to prehospital choking, where the typical anamnestic setting (e.g., onset during a meal, or kids having played with small objects) and the sudden onset of coughing are directing towards the correct diagnosis of a foreign body airway obstruction, both aspects are not valid in the postoperative setting and thus the correct diagnosis may be more difficult.

Concerning the question of how such a large bone fragment could remain within the airway and not being scavenged by the pharynx tampon, we speculate that the bone fragment's shape allowed wedging in situ (explaining the initially frustrated coughing and throat pain of the patient) or that the bone stuck together with blood in situ (explaining the covering blood clot simultaneously expelled with the bone fragment).

Clearly, attempting removal of the foreign body is pivotal to relieve airway obstruction once the diagnosis appears likely, either after direct visualisation (e.g., by a Magill-type forceps) or indirectly, applying endoscopy. Alternative rescue methods to support removal of the obstructing foreign airway body, such as the Heimlich manoeuvre [6] or the Table manoeuvre [7], whereby external force is applied to the thorax, appear to be prone for complications and should be restricted to cases of severe airway obstruction where the patient is unable to breathe or cough and when an immediate direct removal appears impossible.

In summary, this case demonstrates that the differential diagnosis of postoperative airway obstructions should include foreign bodies derived from surgery, including tissue and bone fragments.

## Figures and Tables

**Table 1:** 

Parameter	Bone fragment in situ	Bone fragment expelled
Mental state	Agitated	Cooperative
Patient's position	Upright preferred	Regular
Respiration	Coughing	Normal breathing
O_2_ flow [l/min]	9	3
RF [/min]	20	15
SpO_2_ [%]	92%	97%
RR [mmHg]	151/79	121/70
HR [/min]	80 (136)	64

Key variables before (bone fragment in situ, left column) and after the bone fragment were expelled by the patient (right column) in the postoperative period. After expelling the bone fragment, all variables returned towards normal values. In parallel also the oxygen requirement could be markedly reduced. O_2_ flow: oxygen flow via the face mask; RF: respiratory frequency; SpO_2_: pulse oximetric arterial oxygen saturation; RR: arterial blood pressure; HR: heart rate. The heart rate in brackets indicates the peak heart rate recorded during this period.
